# Laparoscopic versus Open Liver Resection: Differences in Intraoperative and Early Postoperative Outcome among Cirrhotic Patients with Hepatocellular Carcinoma—A Retrospective Observational Study

**DOI:** 10.1155/2014/871251

**Published:** 2014-12-04

**Authors:** Antonio Siniscalchi, Giorgio Ercolani, Giulia Tarozzi, Lorenzo Gamberini, Lucia Cipolat, Antonio D. Pinna, Stefano Faenza

**Affiliations:** ^1^Division of Anesthesiology, Alma Mater Studiorum University of Bologna, Policlinico S. Orsola-Malpighi, Via Massarenti 9, 40138 Bologna, Italy; ^2^Division of Surgery and Transplantation, Policlinico S. Orsola-Malpighi, University of Bologna, Via Massarenti 9, 40138 Bologna, Italy

## Abstract

*Introduction.* Laparoscopic liver resection is considered risky in cirrhotic patients, even if minor surgical trauma of laparoscopy could be useful to prevent deterioration of a compromised liver function. This study aimed to identify the differences in terms of perioperative complications and early outcome in cirrhotic patients undergoing minor hepatic resection for hepatocellular carcinoma with open or laparoscopic technique*. Methods.* In this retrospective study, 156 cirrhotic patients undergoing liver resection for hepatocellular carcinoma were divided into two groups according to type of surgical approach: laparoscopy (LS group: 23 patients) or laparotomy (LT group: 133 patients). Perioperative data, mortality, and length of hospital stay were recorded.* Results.* Groups were matched for type of resection, median number of nodules, and median diameter of largest lesions. Groups were also homogeneous for preoperative liver and renal function tests. Intraoperative haemoglobin decrease and transfusions of red blood cells and fresh frozen plasma were significantly lower in LS group. MELD score lasted stable after laparoscopic resection, while it increased in laparotomic group. Postoperative liver and renal failure and mortality were all lower in LS group.* Conclusions*. Lower morbidity and mortality, maintenance of liver function, and shorter hospital stay suggest the safety and benefit of laparoscopic approach.

## 1. Introduction 

Hepatocellular carcinoma (HCC) is one of the most common malignancies of western countries with an increased incidence in patients with chronic liver disease, especially those with HCV and HBV related cirrhosis [1, 2]. Hepatic resection is certainly one of the most important treatment options and surgery can be carried out with open or laparoscopic technique; among advantages of laparoscopic surgery there are reduction of perioperative bleeding, lower hemodynamic impact, shorter hospital stay, lower postoperative pain, and earlier return to normal activities [3]. However, laparoscopy is considered risky in cirrhotic patients because of the presence of complications related to the disease such as hyperdynamic circulation, coagulopathy, renal failure, and increased intra-abdominal pressure, so its use is widely debated; moreover, some studies report a greater impairment of oncologic integrity, uncontrollable bleeding, and risk of embolism [4]. This study aimed to identify the differences in terms of intraoperative and postoperative complications and early outcome in cirrhotic patients undergoing minor hepatic resection for hepatocellular carcinoma with open or laparoscopic technique.

## 2. Methods

### 2.1. Study Design

After Institutional Review Board approval (Protocol Code: LAPARO2011), this retrospective observational study included a total of 194 cirrhotic patients, undergoing minor hepatic resection for HCC between 2005 and 2010 at the Department of Surgery and Transplantation of the University of Bologna, divided into two groups on the basis of the kind of surgery carried out: laparotomic (LT group) or laparoscopic (LS group) liver resection.

Liver resections were defined according to the Brisbane 2000 classification. Atypical resections were generally reserved for small peripheral lesions, whereas anatomical resections were performed when it was deemed that hepatic functional reserve was sufficient [2, 5].

Indications for liver laparoscopic resection were patients with small number of lesions (≤3), 5 cm or less, located in liver segments 2 to 6 [6].

Patients with tumors which were either large (>5 cm), central, bilateral, or connected with liver hilum, major hepatic veins, or the inferior vena cava were not accepted as candidates for laparoscopic resection (modified from Louisville Statement [7]).

Patients whose lesion characteristics were incompatible with a laparoscopic approach were excluded from the open group.

*(i) LS Group*. It involved 33 patients operated on between 2006 and 2010. Five patients were subsequently excluded for lack of data and other 5 for the conversion into open technique. Causes of conversion were uncontrolled bleeding (2), hepatomegaly (1), and inability to view or isolate the tumor due to technical reasons (2).

The final number of patients included in this group was 23.

For lesions located in the left lobe requiring left lateral lobectomy or wedge resection, the standard three-trocar technique was used.

For lesions located in the other hepatic segments, a four-trocar technique was applied. In all cases, intraoperative laparoscopic ultrasound was applied to confirm the number, size, and location of nodules and guide the extension of the partial hepatectomy. Parenchymal dissection was performed by harmonic scalpel. The main vascular and biliary pedicles were secured with EndoGIA; smaller pedicles were closed by the use of haemoclip. Pringle maneuver was never applied in this series. A closed system suction drain was left in place for 2 days if no major bleeding or postoperative biliary fistula appeared. Fascial closure was attempted only at the umbilical cannula site. If the tumor size was larger than 3 cm, a Pfannenstiel incision was used to take out the piece.

*(ii) LT Group*. It included 161 patients. Twenty-eight of these were later excluded because they underwent major liver resection such as right hepatectomy (9), left hepatectomy (11), enlarged left hepatectomy (4), mesohepatectomy (1), and trisegmentectomy (3).

The final number of patients included in this group was 133.

The right subcostal incision extended to the xiphoid process along the middle line (“J shape incision”) is our standard laparotomy for hepatobiliary surgery. Parenchymal transaction was carried out by harmonic scalpel (similar to the laparoscopic technique) or traditional Kellyclasia technique (based on single surgeon's preference). Vascular clamping was usually applied when excessive bleeding was encountered during transaction; type of clamping was arbitrarily chosen by the operator. Precise detailes about our surgical technique have been already reported [8].

Intraoperative ultrasound was performed to guide all types of liver resections. One or two closed system suction drains were left in place for 48 hours.

Patients in both groups underwent minor liver resection: wedge resection, segmentectomy, bisegmentectomy, or left lobectomy (Table 1), according to IHPBA classification [9].

### 2.2. Anesthesiologic Management

In both groups, induction was performed by intravenous infusion of propofol (1–1.5 mg kg^−1^), fentanyl (3 mcg kg^−1^), midazolam (0.05 mg kg^−1^), and atracurium (0.1 mg kg^−1^) at 100% of FiO_2_. After intubation, maintenance was achieved with sevoflurane (0.8–1.2%), fentanyl, and vecuronium. Mechanical ventilation was performed by setting the tidal volume to 8–10 mL kg^−1^ with 8–12 breaths per minute and a FiO_2_ of 40–50%. In all patients, radial artery and right internal jugular vein were cannulated for monitoring and rapid infusion of liquids. From incision to complete resection, fluid infusions were restricted in order to maintain low central venous pressure values to avoid excessive bleeding during resection.

### 2.3. Statistical Analysis

Statistical analysis was performed using Microsoft Excel 2011 and SPSS 17.0. Preoperative and postoperative variables were compared using *t*-test for numeral variables, Fisher exact test for dichotomous nominal variables, Pearson chi-square test for multicategorical nominal variables, and Mann-Whitney test for ordinal variables. For the analysis of intraoperative variables, we used a two-level linear model for repeated measures using the group membership as a factor between the subjects. All the results with *P* < 0.05 were considered statistically significant. Numeral variables are expressed as mean ± standard deviation (SD), while nominal or ordinals are expressed as a percentage and an absolute value.

## 3. Collected Data

### 3.1. Preoperative Phase

Clinical and biochemical variables recorded in all patients were age, gender, aetiology of cirrhosis (alcohol, HCV, HBV, and HDV), presence of comorbidities (HIV, diabetes) or complications related to cirrhosis (preoperative ascites, esophageal varices), necessity of drug treatment, or portal vein embolization (PVE).

We also evaluated renal and liver function tests, cell blood count and biochemical parameters.

The severity of cirrhosis was assessed by calculating the “Model of End Stage Liver Disease” (MELD) score and Child-Pugh-Turcotte score (CPT), using the latest laboratory data prior to surgery. We also evaluated the total number of HCC nodules (HCC) radiologically detected, the diameter of the lesion (cm), the number of liver segments treated, and any associated procedures.

The two groups were matched based on type of resection (all patients included in the study received minor hepatectomies) and on median number and median diameter of largest nodule. As reported in Table 1, the two groups were comparable for type of resection and tumor characteristics.

### 3.2. Intraoperative Phase

Blood gas analyses were carried out in all patients from the beginning to the end of the resection. We considered for each patient only the baseline and intraoperative worst values. Blood gas parameters recorded, corrected for body temperature and FiO_2_, were as follows.

- Respiratory parameters: oxygen partial pressure (PaO_2_) and carbon dioxide partial pressure (PaCO_2_).
- Metabolic parameters: pH, bicarbonate ions concentration (HCO_3_^−^), bases excess (BE), and haemoglobin (Hb). Other intraoperative variables recorded were amount of blood red cells and fresh frozen plasma transfusions and number of patients transfused, volume of fluids infused (crystalloids, colloids), amount of albumin administered, need for correction of metabolic acidosis with sodium bicarbonate (NaHCO_3_), and hypotensive episodes (systolic blood pressure under 80 mmHg) and their duration. For patients undergoing open resection, we also collected data on the type of clamping and its duration, while length of pneumoperitoneum was recorded for patients undergoing laparoscopic resection.

### 3.3. Postoperative Phase

We recorded worst levels of creatinine, bilirubin, and INR in the first 7 days after surgery, to recalculate MELD score. We recorded also the occurrence of postoperative ascites, bilirubin levels above 3 mg dL^−1^, 50-50 criterion (bilirubin > 50 mmol L^−1^ (>3 mg dL^−1^) and PT < 50%) [10], postoperative coagulopathy (INR > 1.50 or PT < 50% requiring correction with plasma), postoperative renal failure (creatinine > 2 mg dL^−1^, urea > 200 mg dL^−1^), progressive liver failure that caused patient death or needed liver transplantation (OLT), other morbidities, overall mortality and cause of death, and hospital stay (calculated from the first day after surgery until date of discharge).

## 4. Results

Clinical variables, comorbidities, and complications related to cirrhosis are recorded in Table 2; LT patients were significantly older and presented a lower incidence of alcoholic cirrhosis.

Preoperative data are recoded in Table 2; LS group had significantly higher mean INR values and lower alanine aminotransferase (ALT), platelets, and haematocrit, and MELD score was comparable between the two groups. Intraoperative respiratory and metabolic parameters are recoded in Table 3; among metabolic parameters, only Base Excess (BE) showed no significant differences between the two groups (*P* = 0.595) while the other parameters such as pCO_2_ and pH were statistically significant but not clinically salient because they reasonably derived from pneumoperitoneum physiopathology. Operative time was slightly superior in the laparoscopic group.

Intraoperative values of haemoglobin (Hb) also revealed significant differences between the two groups (*P* < 0.001); in fact, haemoglobin remained stable during laparoscopy while it decreased during open resections (Table 3).

There were not any red blood cells (RBC) transfusions in LS group while 27.4% of patients that underwent open resection needed blood transfusions (*P* = 0.002). With regard to transfusions of fresh frozen plasma (FFP), LS group received an average of 0.22 ± 0.52 units of plasma versus 0.68 ± 1.08 units in control group (*P* = 0.02).

Duration of vascular pedicle clamping during laparotomic liver resections was 5–63 minutes; pneumoperitoneum during laparoscopic resections ranged from a minimum of 55 minutes to a maximum of 230 minutes.

Pathologic characteristics of the tumor and the rate of curative resection were comparable between the two groups as reported in Table 4.

Postoperative data are recorded in Table 5.

Worst postoperative serum creatinine was statistically different (*P* < 0.0001) and so also postoperative MELD scores (*P* = 0.035). In 8 patients (34.8%) belonging to the LS group, the 50-50 criterion were fulfilled, while only 25 (12.9%) of the patients in LT group had this criterion satisfied (*P* = 0.014).

Treatment for postoperative liver failure, consisting essentially of liver transplantation (OLT), was applied in 2,3% of patients of LT group and none in LS group (*P* = 0.475).

Although hospital stay was not statistically different (*P* = 0.339), it was smaller in LS group, with a range of 3–29 days and an average duration of 7.61 days, while it was greater in LT group with a mean value of 14.38 days and a range of 4–166 days.

Postoperative mortality was lower in LS group (none), while it reached a value of 7.5% (10 patients) in LT group (*P* = 0.36) (Table 5). The largest part of deaths was caused by progressive liver failure (11 patients) and acute myocardial infarction (2 patients).

## 5. Discussion

To date, no randomized clinical trial evaluating laparoscopic against laparotomic liver resection has still been performed, but case series and prospective studies assessed its safety in terms of oncological integrity and the possibility to reduce postoperative hospital stay [11]. This study focused on perioperative period of cirrhotic patients undergoing minor liver resection and its results are in line with literature [12]; interestingly we found a lower incidence of acute kidney injury and postoperative liver failure which could account for the lower hospital stay underlined in other studies [13].

Minor surgical trauma and a more precise haemostasis, associated with the effect of hydrostatic pressure of pneumoperitoneum on the surface of dissected parenchyma, justify the maintenance of haemoglobin almost constant during surgery, resulting in a reduced necessity for transfusion of blood products. This is an important aspect since bleeding and consequently blood transfusions are associated with a higher postoperative morbidity in cirrhotic patients [11, 13, 14].

In this series we have applied Harmonic scalpel as our basic transaction technique; in the case of laparoscopic major hepatectomies we have used ultrasonic dissector. Anyway, Gobardhan et al. [15] have recently reported that no single method for parenchymal transaction has proven to be better than the other one. Based on single surgeon's experience, ultrasound dissector or ultrasound scissors (such as Harmonic scalpel or Thunderbeat) are mainly used together with monopolar or bipolar coagulators. Stappler technique has been also reported as alternative technique [16].

Postoperative values of MELD scores, expression of an increased risk of hepatic injury [17], were significantly lower after surgery in patients undergoing laparoscopic resection.

Postoperative ascites had a lower incidence in the LS group, and even if this parameter is not statistically significant, it may be an important aspect of postoperative trend. Ascites, in fact, represents a frequent complication of liver resection. Reduction of its incidence in patients undergoing laparoscopic resection can be attributed to the type of surgical approach [18]. This phenomenon could be due to the preservation of collateral circulation in the abdomen, avoiding long incisions and reducing the damage to muscle and round ligament, which may contain important collateral vessels. Other mechanisms that could be part of this phenomenon are the smaller mobilization and manipulation of the liver parenchyma, which reduces the trauma [18], the reduced section of lymph vessels, and minor demand for intraoperative fluids.

Due to these reasons, a reduced incidence of postoperative ascites in resected patients by laparoscopic approach has been reported in particular in F4-liver cirrhosis [19]; similar results have been confirmed in a recent meta-analysis regarding laparoscopic versus open approach for HCC and including 550 patients [20].

Absence of postoperative liver failure in LS group could also be traced in part to this type of mechanisms. This last parameter is an important result; liver failure in fact is one of the most severe postoperative complications, especially in cirrhotic patients, in which this is still more common [21].

The group undergoing laparoscopic liver resection did not develop renal failure. This finding, not underlined from other studies, could be related to lower incidence of ascites [20] and absence of liver failure, which allowed the maintenance of adequate renal function; on the other hand LT group had an incidence of postoperative renal failure of 6.8%.

The reduced incidence of postoperative complications after laparoscopic liver resection for HCC compared to conventional approach has been clearly reported in the literature both by single-center experience and by meta-analysis [13, 19, 20, 22]; we think the reduced functional reserve of cirrhotic patients considered in this study could rapidly impair under intense surgical insults as open surgery.

We have also to underline the conversion rate of laparoscopy (17,9%), which is in line with data found in literature [1]. The main causes of conversion were excessive bleeding and the inability to view/isolate the lesion, in particular at the beginning of our experience. This reflects the fact that laparoscopic liver resection techniques are not a completely solved problem, especially in cirrhotic patients [1]. Certainly, as stated by many authors [11, 14, 23], this procedure should be practiced by experienced surgeons, with an extensive experience in both types of resection and advanced laparoscopy in specialized centres.

Limitations of this study are the important difference in sample size between the two groups and its retrospective nature; another possible limitation could be represented by a significant difference in the average age of the two groups, lower in the LS group. However, homogeneity of the sample in relation to the type of resection, tumor characteristics, underlying liver disease, and other comorbidities, such as diabetes, HIV, preoperative ascites, and esophageal varices, can make this difference less significant.

## 6. Conclusions

Our study showed that intraoperative bleeding and transfusion requirements were significantly lower in the group undergoing laparoscopic liver resection.

Laparoscopic approach, opposed to open surgery, has not led to the development of hepatic or renal failure and mortality, morbidity, and postoperative hospital stay were lower.

The advantages of laparoscopic liver resection compared to traditional technique are several, especially if laparoscopic approach is used in patients with a higher MELD score and with a potentially increased risk of perioperative complications.

## Figures and Tables

**Table 1:** The two groups (laparoscopic versus open resection) were matched based on type of resection and median number and diameter of nodules.

Variables	LS group (23)		LT group (133)		
	Mean	SD	Mean	SD	*P*

Preoperative data					
Number of lesions	1.23 (1–3)	0.56	1.26 (1–6)	0.68	0.898
Diameter of the lesion (cm)	3.21 (1.9–8)	1.04	3.6 (1–9)	1.64	0.074

	Number	%	Number	%	*P*

Type of resection					
Wedge (*n*)	10	43.5%	90	67.7%	0.165
Segmentectomy (*n*)	8	34.8%	27	20.3%	
Bisegmentectomy (*n*)	2	8.7%	8	6.0%	
Left lobectomy (*n*)	3	13%	8	6.0%	

**Table 2:** Preoperative data, clinical variables, comorbidities, and complications related to cirrhosis divided according to type of resection.

Variables	LS group (23)		LT group (133)		
	Mean	SD	Mean	SD	*P*

Age (*n*)	57.91 (30–73)	10.92	63.26 (41–77)	7.89	0.033^*^
Male/female	15/8		104/29		0.19

	Number	%	Number	%	*P*

Pathologies					
Alcohol (*n*)	5	21.7%	6	4.5%	0.011^*^
HCV (*n*)	17	73.9%	88	67.6%	1
HBV (*n*)	6	26.1%	35	26.5%	1
HDV (*n*)	1	4.3%	5	3.8%	1
HIV (*n*)	3	13%	19	14.4%	1
Diabetes (*n*)	4	17.4%	36	27.3%	0.441
Preoperative ascites (*n*)	3	13%	8	6.1%	0.211
Esophageal varices (*n*)	13	56.6%	40	34.8%	0.062

	Mean	SD	Mean	SD	*P*

Preoperative data					
Serum urea (g/L)	0.37	0.12	0.4	0.17	0.521
Serum creatinine (mg/dL)	0.9	0.2	0.98	0.29	0.197
Proteins (g/dL)	7.59	0.71	7.57	0.6	0.878
Albumin (g/dL)	3.91	0.39	3.73	0.41	0.139
Bilirubin (mg/dL)	1.45	1.18	0.98	0.51	0.074
ALP (U/L)	305.5	103.65	281.55	108.28	0.597
AST (U/L)	60	30.84	86.78	77.08	0.103
ALT (U/L)	55.48	43.38	87.76	72.41	0.04^*^
INR	1.25	0.17	1.16	0.11	0.015^*^
MELD	10.09 (7–18)	3.04	8.95 (6–15)	1.82	0.096
Na^+^ (mmol/L)	140.65	3.5	138.8	2.67	0.023^*^
WBC (U/mL)	5272.61	2125.79	5389.83	1728.14	0.772
Hb (g/dL)	13.36	1.74	13.9	1.47	0.119
HCT (%)	39.2	4.37	41.17	4	0.035^*^
PLT (U/mL)	111 217	60 336	138 439	59 880	0.046^*^

^*^ *P* < 0.05.

**Table 3:** Intraoperative respiratory and metabolic parameters, transfusions, and operative time divided in two groups according to type of surgical approach; RBC: red blood cells; FFP: fresh frozen plasma; PT RBC/FFP: patients transfused with units of RBC/FFP.

Variables	LS group (23)		LT group (133)		
	Intraoperative data				

	Mean	SD	Mean	SD	*P*

Basal PaO_2_ (mmHg)	253.02	109.88	227.29	66.57	<0.001^*^
Worst PaO_2_ (mmHg)	181.53	75.86	235.29	68.77	<0.001^*^
Basal PaCO_2_ (mmHg)	34.94	5.07	35.74	4.8	<0.001^*^
Worst PaCO_2_ (mmHg)	43.75	8.56	35.96	4.65	<0.001^*^
Basal pH	7.44	0.04	7.43	0.05	<0.001^*^
Worst pH	7.35	0.07	7.39	0.06	<0.001^*^
Basal HCO_3_^−^ (mmol/L)	23.38	2.46	24.04	1.73	<0.001^*^
Worst HCO_3_^−^ (mmol/L)	23.65	2.37	21.93	1.97	<0.001^*^
Basal BE (mmol/L)	−0.49	2.05	−0.61	2.06	0.595
Worst BE (mmol/L)	−2.3	2.53	−2.69	2.56	0.595
Basal Hb (g/dL)	11.96	1.62	12.23	1.58	<0.001^*^
Worst Hb (g/dL)	12.11	1.84	11.21	1.7	<0.001^*^
RBC (U)	0	0	0.58	1.1	<0.001^*^
FFP (U)	0.22	0.52	0.68	1.07	0.002^*^

	Number	%	Number	%	*P*

PT RBC	0	0%	36	27.4%	0.002^*^
PT FFP	4	17.4%	45	34.2%	0.143
Acidosis correction (NaHCO_3_)	2	8.7%	16	19%	0.35

	Min	SD	Min	SD	*P*

Operative time	175	91	165	80	0.588

^*^ *P* < 0.05.

**Table 4:** Pathologic tumor characteristics and curative resection based on laparoscopic versus open resection.

Variables	LS group (23)		LT group (133)		*P*
	Number	%	Number	%	
Edmonds on grades I-II	8	34.8%	42	31.6%	0.951
Edmonds on grades III-IV	15	65.2%	91	68.4%	

Microvascular invasion (yes)	13	56.5	71	53.4	0.958
Microvascular invasion (no)	10	43.5	62	46.6	

Curative resection (R0)	22	95.6%	129	97.0%	0.761

**Table 5:** Postoperative data: hepatic and renal functions, coagulopathies, other morbidities, mortality, and hospital stay divided in two groups according to type of surgical approach.

Variables	LS group (23)		LT group (133)		
	Postoperative data				

	Mean	SD	Mean	SD	*P*

Worse creatinine (mg/dL)	0.84	0.18	1.18	0.44	<0.001^*^
Worse bilirubin (mg/dL)	2.48	1.45	2.17	3.43	0.673
Worse INR	1.33	0.18	1.33	0.19	0.987
Worse MELD	10.6 (5–17)	3.58	12.6 (5–29)	4.24	0.035^*^

	Number	%	Number	%	*P*

PO ascites	3	13%	34	25.8%	0.288
PO jaundice	10	43.5%	30	22.7%	0.067
50-50 criterion	8	34.8%	17	12.9%	0.014^*^
PO coagulopathy	5	21.7%	25	18.9%	0.777
PO renal failure	0	0%	9	6.8%	0.357
POLF	0	0%	9	6.8%	0.357
Treatment of POLF	0	0%	3	2.3%	0.475
Morbidity	5	21.7%	47	35.6%	0.237
Status (dead/alive)	0/23	0/100%	10/123	7.5/92.5%	0.36

	Mean	SD	Mean	SD	*P*

Hospital stay (*n*)	7.61 (3–29)	5.5	14.38 (4–166)	21.78	0.339

^*^ *P* < 0.05.
